# Socioeconomic Inequalities in Out-of-Pocket and Catastrophic Health Expenditures in Pakistan

**DOI:** 10.3389/ijph.2024.1607313

**Published:** 2024-11-12

**Authors:** Saima Bashir, Shabana Kishwar, Muhammad Nasir, Shehzad Ali

**Affiliations:** ^1^ Manchester Centre for Health Economics (MCHE), The University of Manchester, Manchester, United Kingdom; ^2^ Centre for Business & Economic Research (CBER), School of Economics & Social Sciences (SESS), Institute of Business Administration, Karachi, Sindh, Pakistan; ^3^ Western Centre for Public Health & Family Medicine, Western University, London, ON, Canada

**Keywords:** socio-economic inequalities, poverty, Pakistan, catastrophic health expenditures, out of pocket health expenditures

## Abstract

**Objectives:**

In Pakistan, healthcare utilization is linked to out-of-pocket payments (OOP) which disproportionately affect low-income households. We investigated socioeconomic inequality in OOP and catastrophic health expenditures (CHEs), and the contribution of sociodemographic factors to these inequalities.

**Methods:**

Socioeconomic inequalities were quantified using the concentration index (CI), and the slope (SII) and relative (RII) indices of inequality using data from three rounds of Household Integrated Economic Survey (2007-08, 2011-12, and 2018-19). Decomposition analyses were conducted using the Wagstaff and Erreygers approach.

**Results:**

OOP payments increased from PKR 127 (2007-08) to PKR 250 (2018-19). CHEs in the most deprived quintile (Q1) changed from 8.3% (2007-08) to 13.7% (2018-19), and for the least deprived quintile (Q5) from 5.1% (2007-08) to 8.4% (2018-19). The OOP CI increased from 0.028 to 0.051, while the SII and RII increased from 0.89 to 1.32 and 1.18 to 1.36, respectively. Decomposition analysis showed that household size, composition, employment, and the province of residence explained much of the socioeconomic inequality in CHEs.

**Conclusion:**

Poor households experience high CHE, disproportionately impacting larger families with children and elderly members. Policymakers should implement targeted financial protection strategies to safeguard vulnerable households from the impoverishing effects of healthcare expenses.

## Introduction

Achieving universal health coverage, including financial protection, is a fundamental right and a key Sustainable Development Goal (SDG 3.8). Across the world, households face severe financial challenges in accessing healthcare [1]. This was exacerbated by the COVID-19 pandemic which reduced households’ ability-to-pay for care, resulting in significant inequities in the impoverishing effect of out-of-pocket (OOP) healthcare expenditure [2].

Poor households are susceptible to experiencing financial hardship due to OOP expenditures because of low levels of disposable income which are largely consumed by essential expenditures [3, 4]. In low middle-income countries (LMICs), OOP expenditures are of particular concern due to limited health insurance coverage [5]. It is, therefore, important to quantify and track the level of and disparity in OOP expenditures and its catastrophic financial impact on households to inform the development of equitable policies and programs that can reduce financial barrier to care-seeking and mitigate the impoverishing effect of OOP payments [6–10].

In a health financing model reliant on OOP payments, the principle of pay-as-you-go results in payments rising in proportion to use. Consequently, households lacking adequate financial safeguards often find themselves depleting their economic reserves, leaving minimal resources for subsistence [4]. When the cost of healthcare becomes disproportionately high relative to financial capacity, the resultant financial strain is termed “catastrophic.” A common ethical standpoint is that individuals should not have to allocate more than a predetermined fraction of their income, wealth, or consumption expenditure [11]. Most people facing catastrophic health expenditures (CHEs) and the impoverishing effects of OOP payments live in LMICs [12]. However, there is significant variation in incidence estimates across studies, based on the population surveyed, study methodology and sampling strategies. For instance, the incidence of CHEs at the 10% threshold for individuals aged 20 to 59 is 17.69% in Afghanistan, 13.8% in Bangladesh and 3.97% in Bhutan [13]. In China and India, the incidence of CHE at 10% threshold was 39.4% [14] and 16.7% [15], respectively. In sub-Saharan Africa, the incidence of CHE has been reported to range between 0.1% and 25.4% across countries, with highest rates reported for Democratic Republic of Congo, Benin and Nigeria [16]. Overall, it is estimated that approximately 1 billion people in the world experienced CHEs in 2019, and over half a billion were pushed below the poverty line due to healthcare payments [1, 2].

In Pakistan, a major proportion of healthcare is financed through OOP payments [8]. In the fiscal year 2019-20, 39.8%% of the total healthcare expenditures was financed by the public sector, whereas 59.7% was sourced from private funding. A substantial portion of this private healthcare expenditure, i.e., 88.6%, comprised OOP health payments [17]. These payments relates to the cost of consultations with healthcare providers, diagnostic investigations, medications, inpatient admissions and surgical procedures. Even if care is sought in public healthcare facilities, patients make significant payments for medications and investigations. Moreover, informal payments, paid in the form of cash, presents or gratitude aids to overcome care-seeking barriers, can make up a significant proportion of the total out-of-pocket costs in Pakistan [18]. Over time, there has been a persistent rise in OOP healthcare spending [17] and CHEs [8], attributable to inadequate government funding, absence of comprehensive financial risk protection schemes, and escalating treatment costs. Due to the expectation of substantial OOP expenses, poor households often defer seeking medical attention until the severity of their illness necessitates more protracted and costly interventions [19]. This contributes to elevated levels of unmet healthcare needs and suboptimal health outcomes. Moreover, in situations where treatment costs are high, economically disadvantaged households frequently turn to informal medical care as a more economical alternative, despite its reduced efficacy and potential adverse impact on health [20], thereby exacerbating existing health disparities.

OOP expenditure tends to increase with income in absolute terms because less deprived households often seek private medical care which tends to have better quality and higher price [21] However, given their baseline income, high OOP payments tend not to be catastrophic as they make up a smaller percentage of income. On the contrary, lower income households might be overburdened by incurring a higher proportion of income as OOP cost, with catastrophic impact on household finances.

Inequalities in the burden of CHEs and disparities in access to and utilization of healthcare services may exacerbate due to socioeconomic differences in OOP expenditures [22]. Previous research consistently demonstrates that CHEs disproportionately affect the poorest segments of society [12, 23, 24]. Studies have revealed, for instance, that the wealthiest households are least affected by CHEs [25–27]. In case of Pakistan, the incidence of CHE was estimated to be 0.45% in 2015-16 which increased to 4.57% in 2018-19, based on a 40% threshold definition of CHE [8]. This represents a significant increase in CHE within a short period of time. Bashir and Kishwar [8] quantified the level of OOP and the incidence of CHE in Pakistan. *However, they* did not investigate socioeconomic inequalities in OOPs and CHEs and the contribution of different explanatory factors to these inequalities; this is the focus of the current study. This study evaluates the levels and changes in socioeconomic inequality in OOPs and CHEs, and investigates the factors influencing CHEs using a decomposition analysis, to guide policymakers to develop specific policies to reduce inequalities.

## Methods

### Data

OOP health expenditures are obtained from three rounds of National Health Accounts (NHA), while individual and household characteristics are obtained from Household Integrated Economic Survey (HIES, 2007-08, 2011-12, and 2018-19) as HIES and NHA surveys have same sample households. The survey used a stratified two-stage sampling design, treating each administrative division in urban and rural domains as separate strata. Primary Sampling Units (PSUs) were selected from each stratum using probability proportional to size method (this sampling method incorporates randomness in the selection process). Households representing Secondary Sampling Units (SSUs) were selected from the sample PSUs, with 12 from urban and 16 from rural domains using systematic sampling technique. The households covered ranges from 15512 in 2007-08 to 24809 in 2018-19. NHA survey provides the detailed expenditures including parchi and admission fees, medicines/vaccine, supplies/medical durables, food, diagnostic tests, doctor and staff, tips, cost of surgery, transportation costs, accompanying person/carer cost, and others.

NHA (2009-10) is the first dedicated survey on private OOP health expenditures. Data on OOP for 2007-08 were extrapolated by using OOP health expenditures data from NHA (2009-10) as a yardstick. This study used NHA survey and estimated OOP health expenditures at household level by aggregating the individual OOP health expenditures of inpatients and outpatient services on different heads. Furthermore, individual and household characteristics including consumption expenditures, family size, number of children, educational attainment, age, marital status, gender, and employment status were obtained from HIES.

### Variables

This study used per adult equivalent OOP health expenditures and CHEs as dependent variables in the analysis. For measuring CHEs, firstly, the ratios of OOP health expenditures to total consumption expenditures/non-food expenditures were estimated. The shares preferred over absolute value of OOP health expenditures as welfare consequence of these payments are clearly depicted. Secondly, CHEs are measured if OOP health expenditures as a proportion of household’s total consumption expenditures exceeds 10% threshold or CHEs out of non-food expenditures exceed 40% threshold [28–30]. The SES (poorest, poor, middle, rich and richest) form poorest to richest presented in the form of five quintiles (Q1 to Q5) is based on per adult equivalent total consumption expenditures of the households. The expenditures of previous rounds are adjusted for 2018-19 prices by using consumer price index (CPI) reported by Word Development Indicators (WDI).

To identify the relevant set of variables to investigate the decomposition analysis, we reviewed previously published studies. Eze et al (2022) conducted a systematic review of CHE in sub-Saharan Africa and identified the following factors to be significantly associated with CHE: household size, socioeconomic status, age, marital status, education, employment status and rural/urban geography [29]. Vahedi et al (2020) reported that household socioeconomic status and size were the key determinants of inequality in CHE in Iran [31]. Akhtar et al (2020) and Sriram et al., (2024) additionally identified regional differences as important determinants of inequality in India [32, 33]. Similarly, in China, Fu (2022) identified that income, household size, education, age and geography were the five largest contributors to inequality in CHE [34]. Informed by these studies, we selected variables for decomposition analysis.

Explanatory variables at household level consist of presence of at least one child (age less than 6 years), one older household member (age greater than 65 years), one employed household member, and household composition consisting of less than 6 members, 6 to 11 members, and greater than 11 members. The dummies of explanatory variables of household head characteristics such as marital, employment and residential status, gender, and different categories of age, education and provinces are also included in the analysis. These variables were employed to examine potential factors influencing OOP health expenditure/CHEs and served as the base for decomposing socioeconomic inequalities [31, 35]. The variables such as age composition of household members underscore the differential need for healthcare expenditure by reflecting the varying demands for medical care among different households. Non-need variables (geographical location, family size, age, gender, marital status, employment status, education, etc.) have significant explanatory power regarding the inequality observed in healthcare expenditures [33].

### Empirical Methodology

#### Measurement of Inequalities

The socioeconomic inequalities in OOP health expenditures and CHEs are measured through Concentration Index (CI), Slope Index of Inequality (SII) and Relative Index of Inequality (RII) [36–38]. The CI is based on the concentration curve (CC) and measures the inequalities in relative terms. The negative value of CI indicates that inequalities in outcome variable are pro-poor, while a positive value of CI indicates that inequalities in outcome variable are pro-rich. Per adult equivalent consumption expenditures are used as a rank variable in the measurement of CI.

A number of indices of inequality exist in the literature. These range from a simple difference between two groups (for instance, the richest and the poorest quintiles) to more complex ordered indices that provide more nuanced and detailed information on inequalities. Ordered measures are divided into two classes: regression-based measures (i.e., slope and relative indices of inequality) and disproportionality measures (i.e., concentration index). The SII and RII measures represent the absolute and relative differences in outcome, respectively, between the most and least advantaged groups while considering the full distribution of outcomes [39]. These measures are sensitive to the average outcome in the population. In comparison, the concentration index uses information on all individuals and estimates overall inequality across the full socioeconomic distribution, as opposed to comparing the two ends of the distribution. Both measures have advantages and disadvantages. SII and RI have the advantage of ease of interpretation because of the point of comparison being two ends of the socioeconomic distribution. However, SII is sensitive to changes in the mean level of population health. RII overcomes this challenge by dividing SII by the mean population health; however, its interpretation may raise difficulty for users who are not accustomed to using such relative indices [40]. CI overcomes many of these challenges but are difficult to interpret without accompanying concentrative curves. For these reasons, we report the analyses using all three measures to facilitate interpretation and to meet the needs of different audiences.

Additionally, the value of CI, in case of ratio scale variable lies between −1 and +1, but when the outcome variable is binary, as is the case for CHE, it lies between µ-1 and µ+1 and is sensitive to the mean value of the variable [41]. Wagstaff (2005) has proposed an alternative approach, i.e., a normalized CI for bounded variables; however, the normalized index does not satisfy the following four properties of CI in case of binary variables: mirror image, cardinality, transferability and the level of independence [42]. Therefore, we used Erreygers (2009) method which satisfies all four properties in case of binary variable [41]. The CI is formally derived as follows.

$$CI=\frac{2}{\mu }cov\;\left(y_{i}r_{i}\right)$$ (1)

Where $r_{i}$ is the fractional rank of ^*i*^ *th* household across SES as measured by consumption expenditure, $y_{i}$ is the health variable of interest which is the incidence of OOP expenditures and *μ* is the mean of $y_{i}$ . For CHEs, Erreygers [41] CI is described as follows (Equation 2);

$$ECI=\frac{4m}{Z^{\max}-Z^{\min}}CI$$ (2)

Where *m* is the mean of CHEs, $Z^{\max}$ and $Z^{\min}$ are the maximum and minimum values (i.e., 1 and 0) of CHEs, respectively and CI is concentration index of CHEs obtained by using Equation 1.

The SII measure inequalities, as described in Equation 3, in absolute terms in outcome variable of the movement from the highest level to the lowest level in SES. It can be expressed as;

$$SII=y\left(1\right)-y\left(0\right)$$ (3)

Where SII stands for slope index of inequality, $y\left(1\right)$ and $y\left(0\right)$ are the predicted outcomes of the individuals/households at the highest and the lowest position in SES, respectively. The RII can be expressed as;

$$RII=y\left(1\right)/y\left(0\right)$$ (4)

Where RII stands for relative index of inequality (Equation 4). The values of this index greater than 1 represent the pro-rich inequality, while a value less than 1 represents the pro-poor inequalities.

#### Decomposition of Concentration Index

The method proposed by Wagstaff, Van Doorslaer [11] is used for decomposing the inequalities in OOP health expenditures, while for decomposing the inequalities in CHEs, Erreygers [41] corrected CI is applied. In decomposition analysis, firstly a health outcome variable $h_{i}$ is regressed on a set of k explanatory factors $x_{k},$ using Equation 5:

$$h_{i}=\alpha +\sum_{k}\beta_{k}x_{ki}+\varepsilon_{i}$$ (5)

Where $\beta_{k}$ is the vector of regression coefficients/marginal impacts obtained through linear and logistic regression, $x_{k}$ is a set of k explanatory variables, and $\varepsilon_{i}$ is random error term. Secondly, Wagstaff CI of $h_{i}$ can be decomposed as follows;

$${WCI}_{h}=\sum_{k}\left({\frac{\beta_{k}\bar{x_{k}}}{\mu }C}_{k}\right)+\frac{{GC}_{\varepsilon }}{\mu }$$ (6)

and Erreygers CI can be decomposed as follows;

$${ECI}_{h}=4\left(\sum_{k}\beta_{k}\left({\bar{x_{k}}C}_{k}\right)\right)+{GC}_{\varepsilon }$$ (7)

Where $W{CI}_{h}$ and ECI are Wagstaff and Erreygers CI of *h*, $\beta_{k}$ are the estimated regression coefficients for each determinant, $x_{k}$ is the mean value of each explanatory variable, $C_{k}$ is the CI for explanatory variable, μ is mean of the *h* and ${GC}_{\varepsilon }$ is the GCI (generalized concentration index) for the error term. The first part in Equations 6, 7 represents explained inequality caused by variation in the explanatory factors, while the second part represents the unexplained inequality. The absolute contribution of each factor to the overall socio-economic inequality in OOP/CHEs can be expressed as follows (Equation 8);

$$W{AC}_{h}=\left({\frac{\beta_{k}\bar{x_{k}}}{\mu }C}_{k}\right)\text{ or }{EAC}_{h}=4\left(\beta_{k}\left({\bar{x_{k}}C}_{k}\right)\right)$$ (8)

The relative contribution is then can be expressed as follows (Equation 9);

$$W{RC}_{h}=\left(\frac{\beta_{k}\bar{x_{k}}}{\mu }\frac{C_{k}}{WCI}\right)\text{ or }{ERC}_{h}=4\left(\sum_{k}\beta_{k}\left(\bar{x_{k}}\frac{C_{k}}{ECI}\right)\right)$$ (9)

## Results

**FIGURE 1:**
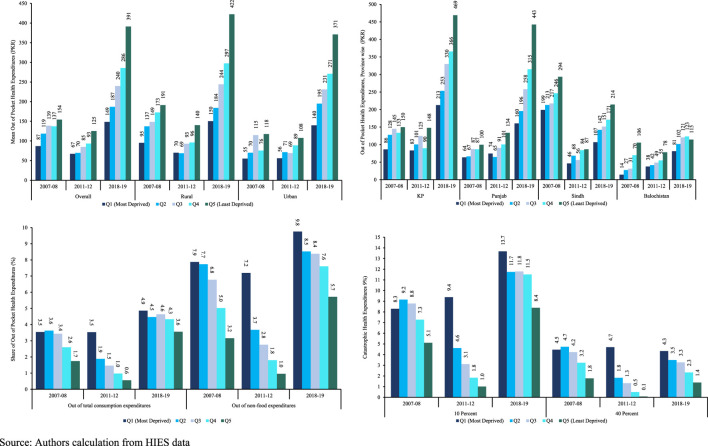
Distribution of Out of Pocket and Catastrophic Health Expenditures in Pakistan, 2007-2019. Source: Authors calculation from HIES data.

The first panel of Figure 1 shows the average distribution of OOP health expenditures across different SES groups at national and regional level. The difference in mean OOP health expenditures of the poorest and richest groups increased by approximately 3.8 times between 2007-08 and 2018-19. This is primarily driven by the increased inequality in mean OOP spending in the urban region where this difference increased by 3.7 times compared to that of 2.8 in the rural region. Moreover, the average OOP expenditures are higher in rural areas compared to urban areas almost across all the income groups.

In 2007-08 and 2018-19, the percentage of households facing CHEs at the 10% threshold was 8.3%, with the incidence higher at 13.7% in the poorest quintile during both periods (panel d, Figure 1). Conversely, in 2007-08, the richest group experienced a CHEs rate of 5.1%, which increased to 8.4% in 2018-19. Examining the 40% threshold, the poorest group exhibited a consistent level of CHEs across the years. In contrast, the CHEs for the richest groups declined over time. Analyzing the 10% and 40% thresholds reveals interesting dynamics. While the poorest group’s CHE remains relatively stable at the 40% threshold, the richest groups show a decline over time. This may suggest that wealthier households are better positioned to manage higher thresholds of health expenditure without it becoming catastrophic.

**FIGURE 2:**
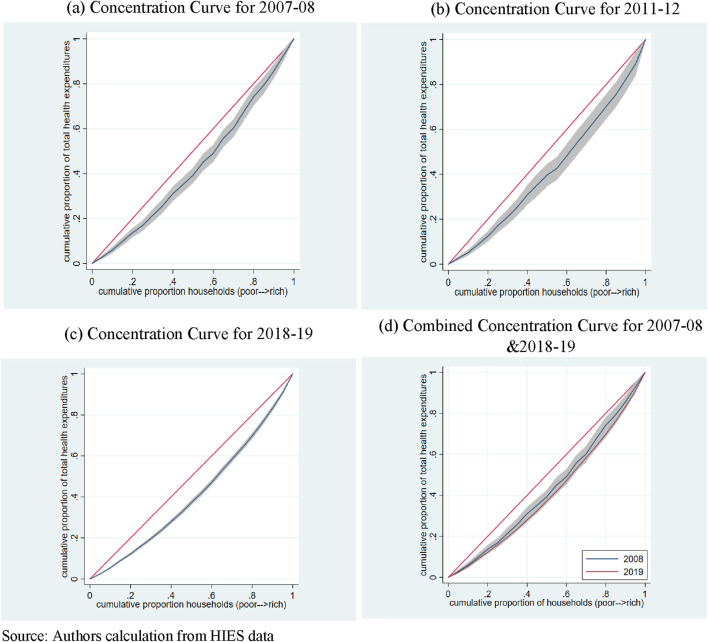
Concentration Curves (Out of Pocket Health Expenditures), Pakistan, 2007-2019. **(A)** Concentration Curve for 2007-08. **(B)** Concentration Curve for 2011-12. **(C)** Concentration Curve for 2018-19. **(D)** Combined Concentration Curve for 2007-08 and 2018-19. Source: Authors calculation from HIES data.

Figure 2 provides a comprehensive view of OOP health expenditures through concentration curves (CCs) in panels a-c, representing the years 2007-08, 2011-12, and 2018-19 respectively. The CC in each case lies below and away from the line of equality, revealing pronounced inequalities in OOP health expenditures across various socioeconomic groups. This positioning underscores a pro-rich distribution of these expenditures.

In panel (d), the concentration curves of 2007-08 and 2018-19 are juxtaposed. The CC for 2018-19 resides below that of 2007-08, indicating an escalation in socioeconomic inequalities in OOP health expenditures over time. However, an examination using the multiple correspondence analysis (MCA) and the intersection union principle (IUP) does not yield statistically significant dominance of one curve over the other, suggesting no significant change in inequality in OOP health expenditures between the years 2007-08 and 2018-19.

**FIGURE 3:**
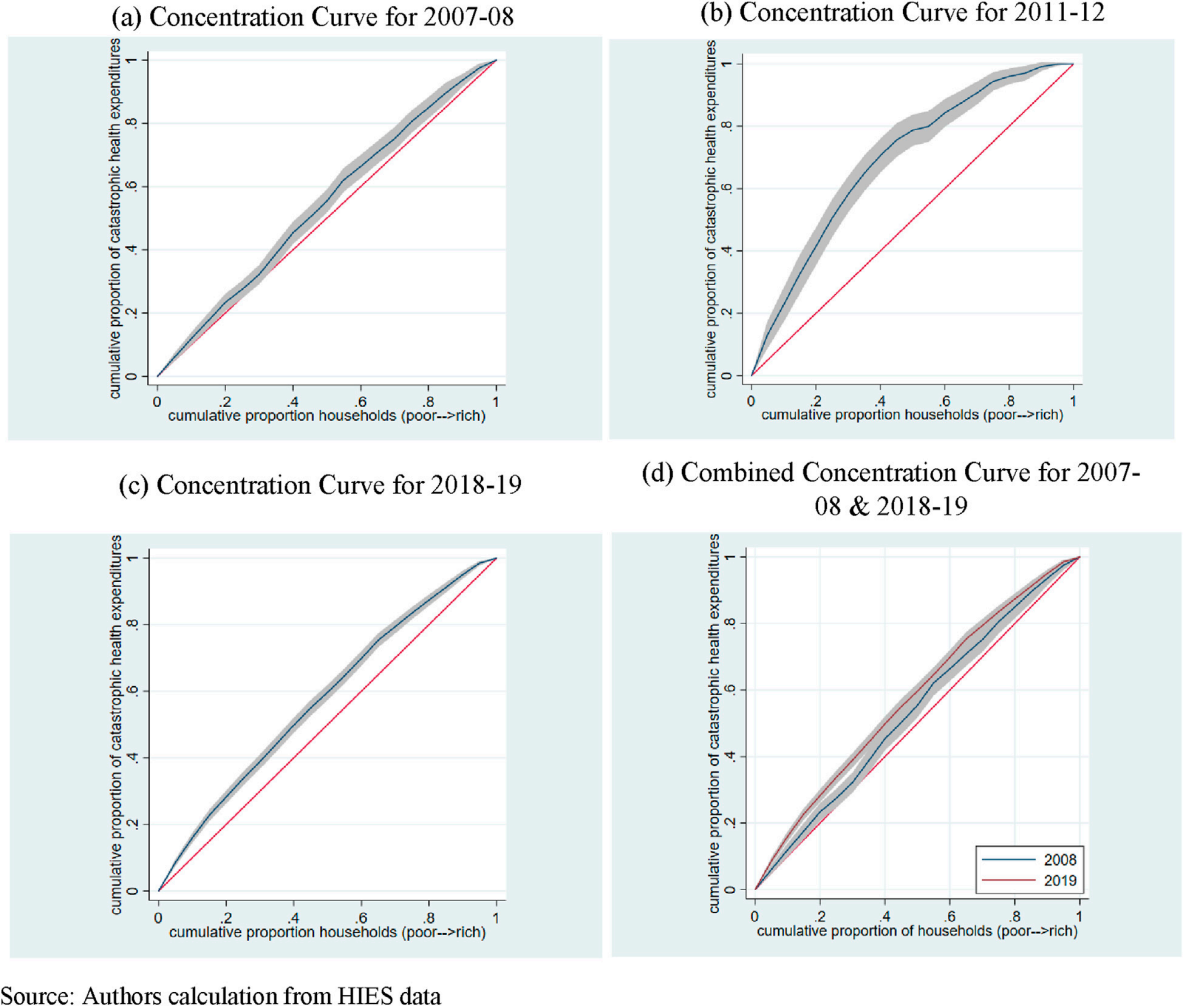
Concentration curves (Catastrophic Health Expenditures). **(A)** Concentration Curve for 2007-08. **(B)** Concentration Curve for 2011-12. **(C)** Concentration Curve for 2018-19. **(D)** Combined Concentration Curve for 2007-08 and 2018-19. Source: Authors calculation from HIES data.

There exist inequalities in CHEs across different SES groups (Figure 3). Additionally, the distribution of CHEs is pro-poor. As the CC of 2018-19 lies above that of 2007-08, the socioeconomic inequalities in CHEs in 2018-19 are higher compared to 2007-08 (panel d). The MCA test of dominance is also found to be significant.

The distribution of households by the various socioeconomic characteristics in 2007-08, 2011-12 and 2018-19 is presented in Supplementary Appendix Table A1. The majority of the households are headed by male, married, employed, uneducated, and age greater than 34 individuals. We also observed that more than 50% of households have children less than 6 years of age and almost 21% households have members older than 65 years of age. However, it is also observed that more than 50% of households are composed of 6–11 members and reside in rural areas.

Table 1 presents comprehensive findings using the CI, SII, and RII. The positive CI for OOP health expenditures indicate a concentration among higher SES groups, implying that the wealthiest individuals allocate a larger portion of their spending towards healthcare services. Both SII and RII values are positive, confirming pro-rich inequalities. Specifically, the SII for OOP health expenditures reveals an increase of 0.89% (2007-08) and 1.32% (2018-19) when transitioning from the lowest to the highest SES group in each respective period. Furthermore, the RII values stand at 1.18 and 1.36 for 2007-08 and 2011-12, respectively. These figures indicate that the probability of incurring OOP health expenditures is higher for the richest groups compared to the poorest group. The upward trend in RII values suggests an ongoing increase in inequalities in OOP health expenditures between the richest and poorest segments of the population.

**TABLE 1:** Concentration index, slope and relative index of inequality (out of pocket and catastrophic health expenditures), Pakistan, 2007-2019.

	OOP	CHE10	CHE40
Concentration index			
	Wagstaff Standard CI	Wagstaff Normalized CI	
2007-08	0.028***(0.003)	−0.09***(0.024)	−0.16 ***(0.031)
2011-12	0.026***(0.004)	−0.42***(0.48)	−0.53*** (0.068)
2018-19	0.051***(0.003)	−0.09***(0.015)	−0.21***(0.025)
		Erreygers CI	
2007-08	---	−0.27***(0.007)	−0.02*** (0.005)
2011-12	---	−0.06***(0.007)	−0.04***(0.005)
2018-19	---	−0.04***(0.006)	−0.03***(0.003)
Slope Index			
2007-08	0.89***(0.107)	−0.04*** (0.012)	−0.03***(0.008)
2011-12	0.69***(0.111)	−0.11***(0.01)	−0.07***(0.011)
2018-19	1.32***(0.070)	−0.06***(0.010)	−0.04***(0.005)
Relative Index			
2007-08	1.18***(0.023)	0.58***(0.00)	0.39***(0.073)
2011-12	1.15***(0.027)	0.06***(0.008)	0.02***(0.008)
2018-19	1.36***(0.023)	0.62***(0.041)	0.30***(0.041)

Note: Standard Error are given in parentheses.

***, **, * represents significance at 1%, 5%, and 10% level of significance.

Total number of observations are 15,323, 15,757, and 24,418 for 2007-08, 2011-12, and 2018-19.

CHE10 and CHE40 represent catastrophic health expenditures at 10% and 40% threshold, respectively.

The negative CI of CHEs states that the poor people are more likely to incur CHEs than the rich. The SII of CHEs shows that while moving from lowest to the highest SES group, the CHEs are decreased by 4% and 6% in 2007-08 and 2018-19 respectively at the 10% threshold. The RII is 0.58 and 0.62 in 2007-08 and 2019-18 respectively, suggesting that likelihood of incurring CHEs of the richest group are lower compared to the poorest group.

### Decomposition Analysis

Tables 2, 3 presents the results of decomposition of CI of OOP health expenditures and CHEs. Columns (3, 7, and 11) represent the CI of each factor, while columns (4, 8, and 12) columns (5, 9, and 13) present the absolute and relative contribution of each factor towards overall inequality in OOP health expenditure/CHEs. The negative sign of CI of a factor implies that the factor is more concentrated amongst the poor; conversely a positive sign shows that the that factor is more concentrated amongst the rich. Absolute contributions measure the share of socioeconomic inequalities in outcome variable that arises from socioeconomic inequalities in a particular factor. A positive absolute contribution indicates that inequalities in outcome variable are concentrated among the rich due to socioeconomic inequalities in the factor. Conversely, a negative absolute contribution suggests that inequalities in outcome variable due to inequalities in a factor are more concentrated among the poor. Relative contribution is the proportion (percentage) of absolute contribution of each factor to overall inequalities in OOP health expenditures.

**TABLE 2:** Decomposition of inequalities in out-of-pocket health expenditures, Pakistan, 2007-2019

	2007-08				2011-12				2018-19			
(1)	(2)	(3)	(4)	(5)	(6)	(7)	(8)	(9)	(10)	(11)	(12)	(13)
	ME	CI	absolute	relative	ME	CI	absolute	relative	ME	CI	absolute	relative
Socioeconomic Quintiles (ref: poorest)												
Poor	0.274***	−0.400	−0.110	−3.356	0.125*	−0.400	−0.050	−1.779	0.19***	−0.400	−0.075	−1.474
Middle	0.382***	0.000	0.000	0.001	0.162**	0.000	0.000	0.000	0.38***	0.000	0.000	0.000
Rich	0.501***	0.400	0.200	6.127	0.133	0.400	0.053	1.901	0.52***	0.400	0.208	4.071
Richest	0.644***	0.800	0.520	15.765	0.47***	0.800	0.374	13.303	0.73***	0.800	0.585	11.434
Household Size (ref: <6 members)												
Members (6–11)	−0.38***	−0.056	0.020	0.652	−0.45***	−0.050	0.020	0.724	−0.26**	−0.080	0.021	0.402
Members (>11)	−0.64***	−0.284	0.180	5.529	−0.61***	−0.120	0.071	2.544	−0.52***	−0.310	0.159	3.112
Household composition												
At least one child (<6 years)	−0.079	−0.124	0.010	0.298	−0.13**	−0.070	0.009	0.320	0.058**	−0.128	−0.008	−0.147
At least one member (>=65 years)	0.147**	−0.045	−0.010	−0.204	0.138***	−0.010	−0.001	−0.053	0.11***	−0.028	−0.003	−0.061
At least one employed member	0.044	−0.014	−0.000	−0.019	−0.022	−0.010	0.000	0.008	0.086	−0.016	−0.001	−0.027
Household Head Characteristics												
Age categories (ref: age<=34)												
35-44	−0.024	−0.023	0.001	0.017	−0.144**	−0.020	0.003	0.118	−0.052	−0.045	0.002	0.046
45-54	0.090	0.015	0.001	0.042	−0.194***	0.030	−0.005	−0.194	−0.035	0.036	−0.001	−0.024
>54	0.070	0.004	0.000	0.008	−0.017	0.010	0.000	−0.007	0.068	0.042	0.003	0.056
Educational Categories (ref: illiterate)												
Primary	−0.194	−0.046	0.009	0.270	0.063	−0.065	−0.004	−0.146	0.024	−0.070	−0.002	−0.033
Metric	−0.269	0.166	−0.045	−1.369	0.116**	0.076	0.009	0.317	0.048	0.144	0.007	0.134
Graduation	−0.255	0.442	−0.113	−3.448	0.225***	0.260	0.059	2.088	−0.060	0.439	−0.026	−0.511
Postgrad	−0.271	−0.158	0.043	1.309	0.290**	0.429	0.125	4.434	−0.049	0.652	−0.032	−0.623
Gender (ref: male)												
Female	−0.226*	−0.011	0.003	0.079	0.130	−0.011	−0.001	−0.052	−0.047	0.178	−0.008	−0.164
Marital Status (ref: unmarried)												
Married	0.116	−0.009	−0.001	−0.033	0.064	−0.007	0.000	−0.016	0.14***	−0.011	−0.002	−0.029
Employment (ref: unemployed)												
Employed (HH)	0.099	−0.011	−0.001	−0.034	−0.142*	−0.016	0.002	0.079	−0.13***	−0.021	0.003	0.053
Region (ref: rural)												
Urban	−0.147**	0.173	−0.025	−0.777	−0.076	0.153	−0.012	−0.414	0.212	0.247	0.052	1.023
Province (ref: KP)												
Punjab	0.253***	−0.041	−0.010	−0.319	−0.040	0.025	−0.001	−0.036	−0.79***	0.097	−0.076	−1.491
Sindh	0.372***	−0.042	−0.016	−0.480	−0.456***	−0.040	0.018	0.647	−1.73***	−0.046	0.080	1.564
Balochistan	−0.136	−0.447	0.061	1.862	−0.362***	−0.134	0.049	1.730	−2.49***	−0.354	0.879	17.183
Residual			−0.684	−20.921			−0.689	−24.516			−1.714	−33.492
CI			0.033	1.000			0.028	1.000			0.051	1.000

Standard errors in parentheses; ***p < 0.01, **p < 0.05, *p < 0.1 represents significance at 1%, 5%, and 10% level of significance. ME, CI, are Marginal Effects and Concentration Index of each factor. Absolute and relative are absolute and relative contribution of each factor to inequality.

**TABLE 3:** Decomposition of inequalities in catastrophic health expenditures (10% threshold), Pakistan, 2007-2019

	2007-08				2011-12				2018-19			
(1)	(2)	(3)	(4)	(5)	(6)	(7)	(8)	(9)	(10)	(11)	(12)	(13)
	ME	CI	absolute	relative	ME	CI	absolute	relative	ME	CI	absolute	relative
Socioeconomic Quintiles (ref: poorest)												
Poor	0.000	−0.320	0.000	−0.001	−0.03***	−0.320	0.008	−0.121	−0.03***	−0.320	0.007	−0.173
Middle	−0.009	0.000	0.000	0.000	−0.05***	0.000	0.000	0.000	−0.03***	0.000	0.000	0.000
Rich	−0.024**	0.320	−0.006	0.232	−0.07***	0.321	−0.018	0.280	−0.04***	0.320	−0.011	0.279
Richest	−0.05***	0.640	−0.026	0.986	−0.10***	0.639	−0.049	0.759	−0.08***	0.640	−0.041	1.086
Household Size (ref: <6 members)												
Members (6–11)	−0.013*	−0.142	0.005	−0.171	−0.012***	−0.111	0.003	−0.050	−0.04***	−0.191	0.019	−0.499
Members (>11)	−0.023**	−0.155	0.002	−0.073	−0.012	−0.060	0.000	−0.005	−0.08***	−0.154	0.006	−0.156
Household composition												
At least one child (<6 years)	−0.007	−0.315	0.005	−0.196	−0.009*	−0.177	0.004	−0.061	0.005	−0.321	−0.004	0.099
At least one member (>=65 years)	0.009	−0.046	0.000	0.016	0.009**	−0.011	0.000	0.002	0.006	−0.027	0.000	0.004
At least one employed member	−0.017	−0.053	0.003	−0.127	−0.007	−0.038	0.001	−0.017	0.002	−0.061	−0.001	0.015
Household Head Characteristics												
Age categories (ref: age<=34)												
35–44	0.002	−0.027	0.000	0.003	−0.02***	−0.024	0.000	−0.006	−0.004	−0.047	0.000	−0.006
45–54	0.011	0.016	0.000	−0.007	−0.015***	0.028	0.000	0.007	−0.003	0.037	0.000	0.003
>54	0.013	0.005	0.000	−0.003	−0.005	0.015	0.000	0.001	0.001	0.051	0.000	−0.002
Educational Categories (ref: illiterate)												
Primary	−0.036	−0.030	0.001	−0.027	0.001	−0.042	0.000	0.000	−0.008	−0.045	0.000	−0.006
Metric	−0.040	0.176	−0.008	0.281	0.007	0.084	0.001	−0.010	−0.007	0.163	−0.001	0.032
Graduation	−0.035	0.158	−0.002	0.075	0.017***	0.090	0.001	−0.008	−0.015	0.174	−0.001	0.027
Postgrad	−0.055	−0.302	0.031	−1.180	0.011	0.070	0.000	−0.002	−0.048**	0.079	0.000	0.012
Gender (ref: male)												
Female	0.009	−0.043	−0.001	0.052	−0.004	−0.041	0.001	−0.010	−0.03***	0.055	0.000	0.013
Marital Status (ref: unmarried)												
Married	0.001	−0.034	0.000	0.005	0.003	−0.026	0.000	0.004	0.002	−0.040	0.000	0.008
Employment (ref: unemployed)												
Employed (HH)	0.001	−0.038	0.000	0.004	−0.011***	−0.051	0.002	−0.030	−0.03***	−0.068	0.006	−0.155
Region (ref: rural)												
Urban	−0.03***	0.228	−0.008	0.305	−0.005	0.205	−0.001	0.019	0.013*	0.359	0.007	−0.178
Province (ref: KP)												
Punjab	−0.03***	−0.039	0.001	−0.044	−0.011	0.058	−0.001	0.023	−0.03***	0.209	−0.013	0.336
Sindh	0.05***	−0.023	−0.001	0.023	−0.026***	−0.038	0.001	−0.015	−0.10***	−0.043	0.004	−0.106
Balochistan	−0.10**	−0.086	0.002	−0.063	−0.034*	−0.023	0.000	−0.002	−0.10***	−0.082	0.002	−0.050
Residual			−0.024	0.911			−0.016	0.243			−0.016	0.418
CI			−0.027	1.000			−0.064	1.000			−0.038	1.000

Standard errors in parentheses; ***p < 0.01, **p < 0.05, *p < 0.1 represents significance at 1%, 5%, and 10% level of significance. ME, CI, are Marginal Effects and Concentration Index of each factor. Absolute and relative are absolute and relative contribution of each factor to inequality.

The findings of the decomposition analysis (CI: Column 3, 7, and 11) reveal that the following factors are concentrated among the poor households: large family size, households with at least one child or one older member, young and employed household head, and residence of relatively less developed province (i.e., Balochistan and Sindh) (Table 3). In contrast to this, the richest households have married, relatively older and educated household head.

The results of absolute (Column 4, 8, and 12) and relative contribution (Column 5, 9, and 13) of CI show that larger family size (6–11 members) increases the inequalities (almost 65.2%, 72.4%, and 40.2% in 2007-08, 2011-12, and 2018-19, respectively) in OOP health expenditures, with higher concentration of OOP among the rich. Similarly, household heads with relatively higher education (postgrad) increases the concentration of OOP health expenditures amongst the rich segment of society in 2011-12 in absolute term, while it increases the inequalities by almost 443%. Furthermore, the households headed by a female are increasing the inequality by 7.9% with high concentration among the rich in 2007-08. Conversely, the households whose heads are married, and the aged between 45 and 54 are decreasing the overall inequality by 3% (2018-19) and 19.4% (2011-12), and the OOP health expenditures are concentrated among the poor. Similarly, the older members are increasing the concentration of OOPs among the poor, while decreasing the inequality by 20%, 5% and 6% in 2007-08, 2011-12 and 2018-19, respectively. Among all the factors, households with larger family sizes have higher relative contributions to inequalities in OOP health expenditures.

Table 3 provides insights from the decomposition of the CI for CHEs at a 10% threshold using Erreygers method (Supplementary Appendix Table A2 represents decomposition at 40% threshold). Across all survey rounds, households with higher family sizes, at least one child, at least one older member, employed head and household heads aged between 35 and 44 years, are concentrated among the poorest (CI: Column 3,7, and 11). Conversely, the richest households are headed by female, and relatively higher educated individual.

The results of absolute (Column 4, 8, and 12) and relative contribution (Column 5, 9 and 13) of CI show that larger family size increase the concentration of CHEs amongst the rich group. However, it is reducing the overall inequality (17%, 5%, and 50% in 2007-08, 2011-12 and 2018-19, respectively). Similarly, the presence of children and older members in the households is increasing the concentration of CHEs amongst the rich, while the former is reducing the inequality (−6.1%) compared to latter group (0.2%). Households with employed heads, and of higher age are increasing the concentration of CHEs amongst the rich, whereas decreasing (3% and 15%) the overall socioeconomic inequalities in 2011-12 and 2018-19, respectively. The households with higher educated head are also increasing the concentration of CHEs amongst the rich, while increasing the overall inequalities by 1.2% (2018-19). For sensitivity analysis, we have also applied the Wagstaff decomposition method, and the results are provided in appendices Supplementary Appendix Tables A3, A4 at 10% and 40% threshold respectively.

## Discussion

In low- and middle-income countries (LMICs), CHEs is a critical welfare concern, driven by inadequate public healthcare services, prompting individuals to resort to paid private care for timely and quality healthcare [6, 8]. The absence of standardized risk-pooling mechanisms, particularly for lower-income groups, not only exacerbates their vulnerability but also pushes them into extreme deprivation [43]. This study represents the initial effort to provide evidence on the evolving distribution of socioeconomic inequalities in OOP health expenditures and CHEs over time in Pakistan, shedding light on how the inequalities in different factors contributes to the inequalities in OOP and CHE.

The study’s key findings underscore significant insights. OOP health expenditures are consistently more concentrated among the rich, while CHEs exhibits a pro-poor distribution. This trend remains consistent across all rounds of datasets and various inequality measures (SII, RII, and CI), aligning with similar patterns observed in countries with comparable healthcare structures, such as Iran, Kenya, and Saudi Arabia structure [6, 7, 44].

Decomposition analysis reveals the nuanced factors contributing to inequalities in OOP health expenditures and CHE. Among all the factors, households with larger family sizes, headed by female, employed and highly educated individuals, and the presence of children (in 2011-12) are increasing the overall socio-economic inequality in OOP health expenditures with higher concentration of OOP among the rich. Conversely, the married and highly aged households’ heads, and presence of older member and children (in 2018-19) are decreasing the overall socio-economic inequalities in OOP health expenditures with higher concentration among the poor. These factors are also emerged as major contributors to inequalities in OOP health expenditures [6, 44, 45]. Since higher education of household heads (metric, graduation and postgrad) increases the burden of OOP health expenditures amongst the rich and their relative contribution in inequalities is positive. This may be since those with higher levels of education have access to more up-to-date knowledge on healthy lifestyle choices, and that in turn raises their demand for specialized healthcare services [6, 38].

Family size, presence of children, employed and of higher age [35–44] and educated household heads (in 2011-12) are decreasing the inequalities in CHE with higher concentration of CHE among the rich. These results are aligned with the previous literature [31, 35]. Conversely, the presence of older members are increasing the inequalities in CHE with higher concentration of CHE amongst the rich.

A pivotal finding is the escalating share of OOP health expenditures and socioeconomic inequalities over time, with the burden increasing and inequalities reaching higher levels in the recent 2018-19 dataset compared to earlier rounds (2007-08 and 2011-12). This rise is attributed to the expanding private healthcare sector in Pakistan, leading to an increased reliance on OOP health payments. Unequal access to and utilization of healthcare services further contributes to growing healthcare spending inequalities.

While health insurance is often implemented without targeting specific groups that contribute most to socioeconomic inequalities in health expenditures, this decomposition analysis makes the case for targeted health financing policies. Such targeting may involve insurance premium waivers, reduced co-payment or better coverage for specific groups identified in the decomposition analysis. In this study children and older members are identified as the factor contributing to socioeconomic inequalities, therefore, it is suggested that the focus of financial risk protection should be this group of society.

This study has a number of limitations. First, it does not account for unmet healthcare need due to the unaffordability of care, i.e., OOP and CHE estimates in this study are based on the cost of the care sought by households which may not align with their level of need – this is because households may not seek sufficient quantity and/or quality of care because of limited resources. As a result, although the incidence of CHE is high, these estimates are still conservative. Second, the survey question on OOP health expenditures lacks specificity regarding reimbursements amount from private health insurance, potentially affecting the interpretation of outcomes. Third, self-reported data on household consumption expenditures in the datasets may introduce recall bias, impacting the accuracy of health expenditure estimates. Fourth, information on the source used to pay for healthcare, such as borrowing from relatives, selling assets and cutting other expenses, was not available in the HIES. These variables might have a considerable impact on household’s financial burden and health-seeking behavior. Finally, the data used in this study are based on a cross-sectional design. Although we pooled the data to see inequality trends over time, longitudinal data can provide better picture of changes in socioeconomic inequalities.

### Conclusion

The discernible rise in socioeconomic inequalities in OOP health expenditures and CHEs in Pakistan underscores the inadequacy of existing financial risk protection mechanisms in addressing these disparities. This study’s findings emphasize the critical importance for policymakers to grasp the nuances of socioeconomic factors contributing to inequities in OOP payments and CHEs, facilitating the design and implementation of targeted and effective policies. A pivotal takeaway from the study is the recognition that factors fueling inequality extend beyond the boundaries of the healthcare system. Addressing socioeconomic inequalities requires a collaborative approach, creating synergies and efficiencies across various sectors.
