# Supplementary material for: Socioeconomic Inequalities in Out-of-Pocket and Catastrophic Health Expenditures in Pakistan
Source: Int J Public Health. 2024 Nov 12;69:1607313. doi: 10.3389/ijph.2024.1607313 (PMC11589653; doi:10.3389/ijph.2024.1607313)
Supplement: Supplementary file 1 [file DataSheet1.docx]

**Table A1.** **Socioeconomic characteristics of the households**

|  | 2007-08 | |  | 2011-12 | |  | 2018-19 | |
| --- | --- | --- | --- | --- | --- | --- | --- | --- |
|  | N | % |  | N | % |  | N | % |
| **Quintiles** | | |  |  |  |  |  |  |
| Poorest | 2,720 | 17.8 |  | 2,702 | 17.2 |  | 4,176 | 17.1 |
| Poor | 2,776 | 18.1 |  | 2,920 | 18.5 |  | 4,466 | 18.3 |
| Middle | 2,970 | 19.4 |  | 3,150 | 20 |  | 4,870 | 19.9 |
| Rich | 3,175 | 20.7 |  | 3,236 | 20.5 |  | 5,146 | 21.1 |
| Richest | 3,682 | 24.0 |  | 3,749 | 23.8 |  | 5,760 | 23.6 |
| **Household Composition** | | |  |  |  |  |  |  |
| Members (<6) | 5,437 | 35.5 |  | 5,996 | 38.1 |  | 10,480 | 42.9 |
| Members (6 to 11) | 8,678 | 56.6 |  | 8,610 | 54.6 |  | 12,369 | 50.7 |
| Members (>11) | 1,208 | 7.9 |  | 1,151 | 7.3 |  | 1,569 | 6.4 |
| Children | 8,774 | 57.3 |  | 8,700 | 55.2 |  | 13,494 | 55.3 |
| Older | 3,318 | 21.7 |  | 3,604 | 22.9 |  | 5,072 | 20.8 |
| **Household Head Characteristics** | |  |  |  |  |  |  |  |
| **Age** |  |  |  |  |  |  |  |  |
| <34 | 2,861 | 18.7 |  | 3,138 | 19.9 |  | 5,297 | 21.7 |
| 34 - 44 | 4,399 | 28.7 |  | 4,187 | 26.6 |  | 6,446 | 26.4 |
| 45 - 54 | 3,797 | 24.8 |  | 3,888 | 24.7 |  | 5,990 | 24.5 |
| > 54 | 4,266 | 27.8 |  | 4,544 | 28.8 |  | 6,685 | 27.4 |
| **Education** |  |  |  |  |  |  |  |  |
| Illiterate | 6,858 | 44.8 |  | 6,602 | 42 |  | 10,55 | 43.3 |
| Primary | 2,410 | 15.7 |  | 2,424 | 15.4 |  | 3,847 | 15.8 |
| Metric | 3,787 | 24.7 |  | 4,140 | 26.4 |  | 6,695 | 27.5 |
| Graduation | 1,674 | 10.9 |  | 1,665 | 10.6 |  | 2,485 | 10.2 |
| Postgrad | 594 | 3.9 |  | 872 | 5.6 |  | 765 | 3.1 |
| **Gender** |  |  |  |  |  |  |  |  |
| Female | 1,170 | 7.6 |  | 1,489 | 9.5 |  | 2,266 | 9.3 |
| Male | 14,153 | 92.4 |  | 14,268 | 90.6 |  | 22,152 | 90.7 |
| **Marital Status** |  |  |  |  |  |  |  |  |
| Otherwise | 1,441 | 9.4 |  | 1,600 | 10.2 |  | 2,256 | 9.2 |
| Married | 13,882 | 90.6 |  | 14,157 | 89.9 |  | 22,162 | 90.8 |
| **Employment Status** |  |  |  |  |  |  |  |  |
| Unemployed | 2,651 | 17.3 |  | 3,070 | 19.5 |  | 4,431 | 18.2 |
| Employed | 12,672 | 82.7 |  | 12,687 | 80.5 |  | 19,987 | 81.9 |
| **Region** |  |  |  |  |  |  |  |  |
| Rural | 9,157 | 59.8 |  | 9,038 | 57.4 |  | 15,714 | 64.4 |
| Urban | 6,166 | 40.2 |  | 6,719 | 42.6 |  | 8,704 | 35.7 |
| **Province** |  |  |  |  |  |  |  |  |
| KP | 2,893 | 18.9 |  | 3,264 | 20.7 |  | 4,405 | 18 |
| Punjab | 6,539 | 42.7 |  | 6,881 | 43.7 |  | 11,625 | 47.6 |
| Sindh | 3,729 | 24.3 |  | 4,190 | 26.6 |  | 6,160 | 25.2 |
| Balochistan | 2,162 | 14.1 |  | 1,422 | 9.0 |  | 2,228 | 9.1 |

Note: N represents number of observations (15323, 15757, 24418 in 2007-08, 2011-12 and 2018-19, respectively).

**Table A2: Decomposition of inequalities in catastrophic health expenditures (40% threshold, Erreygers Methods)**

|  | 2007-08 | | | | 2011-12 | | | | 2018-19 | | | |
| --- | --- | --- | --- | --- | --- | --- | --- | --- | --- | --- | --- | --- |
| (1) | (2) | (3) | (4) | (5) | (6) | (7) | (8) | (9) | (10) | (11) | (12) | (13) |
|  | ME | CI | absolute | relative | ME | CI | absolute | relative | ME | CI | absolute | relative |
| Socioeconomic Quintiles (ref: poorest) | | | | |  |  |  |  |  |  |  |  |
| Poor | -0.001 | -0.320 | 0.000 | -0.011 | -0.02*** | -0.320 | 0.004 | -0.115 | -0.009*** | -0.320 | 0.002 | -0.093 |
| Middle | -0.008 | 0.000 | 0.000 | 0.000 | -0.02*** | 0.000 | 0.000 | 0.000 | -0.013*** | 0.000 | 0.000 | 0.000 |
| Rich | -0.018*** | 0.320 | -0.005 | 0.205 | -0.04*** | 0.321 | -0.010 | 0.283 | -0.024*** | 0.320 | -0.006 | 0.254 |
| Richest | -0.039*** | 0.640 | -0.020 | 0.898 | -0.07*** | 0.639 | -0.035 | 1.001 | -0.040*** | 0.640 | -0.020 | 0.834 |
| Household Size (ref: < 6 members) | | | | |  |  |  |  |  |  |  |  |
| Member (6 to 11) | -0.008* | -0.142 | 0.003 | -0.127 | -0.003 | -0.111 | 0.001 | -0.021 | -0.015*** | -0.191 | 0.007 | -0.286 |
| Member (>11) | -0.019** | -0.155 | 0.002 | -0.074 | -0.003 | -0.060 | 0.000 | -0.002 | -0.037 | -0.154 | 0.003 | -0.117 |
| Household Composition |  |  |  |  |  |  |  |  |  |  |  |  |
| At least one child (<6 yrs) | 0.001 | -0.315 | 0.000 | 0.018 | -0.005 | -0.177 | 0.002 | -0.057 | 0.004*** | -0.321 | -0.003 | 0.123 |
| At least one member (>=65 yrs) | 0.008 | -0.046 | 0.000 | 0.016 | 0.002 | -0.011 | 0.000 | 0.001 | 0.001 | -0.027 | 0.000 | 0.001 |
| At least one employed member | -0.016 | -0.053 | 0.003 | -0.140 | -0.011 | -0.038 | 0.002 | -0.047 | -0.010* | -0.061 | 0.002 | -0.096 |
| Household Head Characteristics | | | | |  |  |  |  |  |  |  |  |
| Age categories (ref: age<=34) | | | | |  |  |  |  |  |  |  |  |
| 35-44 | -0.001 | -0.027 | 0.000 | -0.002 | -0.004 | -0.024 | 0.000 | -0.003 | -0.003 | -0.047 | 0.000 | -0.006 |
| 45-54 | 0.014** | 0.016 | 0.000 | -0.010 | -0.005 | 0.028 | 0.000 | 0.004 | -0.007* | 0.037 | 0.000 | 0.011 |
| >54 | 0.012* | 0.005 | 0.000 | -0.003 | 0.005 | 0.015 | 0.000 | -0.003 | -0.002 | 0.051 | 0.000 | 0.006 |
| Educational Categories (ref: illiterate) | | | | |  |  |  |  |  |  |  |  |
| Primary | -0.028 | -0.030 | 0.001 | -0.025 | 0.004 | -0.042 | 0.000 | 0.003 | -0.003 | -0.045 | 0.000 | -0.004 |
| Metric | -0.027 | 0.176 | -0.005 | 0.224 | 0.006* | 0.084 | 0.001 | -0.016 | -0.002 | 0.163 | 0.000 | 0.017 |
| Graduation | -0.027 | 0.158 | -0.002 | 0.068 | 0.007 | 0.090 | 0.000 | -0.007 | -0.004 | 0.174 | 0.000 | 0.012 |
| Postgrad | -0.031 | -0.302 | 0.018 | -0.791 | -0.001 | 0.070 | 0.000 | 0.000 | -0.004 | 0.079 | 0.000 | 0.001 |
| Gender (ref: male) | | | | |  |  |  |  |  |  |  |  |
| Female | -0.005 | -0.043 | 0.001 | -0.037 | -0.005 | -0.041 | 0.001 | -0.020 | -0.008 | 0.055 | 0.000 | 0.005 |
| Marital Status (ref : unmarried) | | | | |  |  |  |  |  |  |  |  |
| Married | 0.001 | -0.034 | 0.000 | 0.007 | 0.010** | -0.026 | -0.001 | 0.026 | 0.010** | -0.040 | -0.001 | 0.060 |
| Employment (ref: unemployed) | | | | |  |  |  |  |  |  |  |  |
| Employed (HH) | 0.005 | -0.038 | -0.001 | 0.026 | 0.002 | -0.051 | 0.000 | 0.007 | -0.006 | -0.068 | 0.001 | -0.050 |
| Region (ref: rural) | | | | |  |  |  |  |  |  |  |  |
| Urban | -0.020*** | 0.228 | -0.006 | 0.266 | -0.009** | 0.205 | -0.002 | 0.071 | -0.005 | 0.359 | -0.002 | 0.101 |
| Province (ref: KP) | | | | |  |  |  |  |  |  |  |  |
| Punjab | -0.019** | -0.039 | 0.001 | -0.031 | -0.004 | 0.058 | -0.001 | 0.015 | -0.008*** | 0.209 | -0.004 | 0.146 |
| Sindh | 0.021*** | -0.023 | 0.000 | 0.012 | -0.02*** | -0.038 | 0.001 | -0.017 | -0.021*** | -0.043 | 0.001 | -0.033 |
| Balochistan | -0.053*** | -0.086 | 0.001 | -0.039 | -0.010 | -0.023 | 0.000 | -0.001 | -0.028*** | -0.082 | 0.001 | -0.022 |
| Residual | . | . | -0.012 | 0.550 | . | . | 0.004 | -0.103 | . | . | -0.003 | 0.135 |
| CI | . | . | -0.022 | 1.000 | . | . | -0.035 | 1.000 | . | . | -0.025 | 1.000 |
| Standard errors in parentheses; *** p<0.01, ** p<0.05, * p<0.1 represents significance at 1%, 5% and 10% level of significance. ME, CI, are Marginal Effects and Concentration Index of each factor. Absolute and relative are absolute and relative contribution of each factor to inequality. | | | | | | | | | | | | |

**Table A3: Decomposition of inequalities in catastrophic health expenditures (10% threshold, Wagstaff Method)**

|  | 2007-08 | | | | 2011-12 | | | | 2018-19 | | | |
| --- | --- | --- | --- | --- | --- | --- | --- | --- | --- | --- | --- | --- |
| (1) | (2) | (3) | (4) | (5) | (6) | (7) | (8) | (9) | (10) | (11) | (12) | (13) |
|  | ME | CI | absolute | relative | ME | CI | absolute | relative | ME | CI | absolute | relative |
| Socioeconomic Quintiles (ref: poorest) | | | | |  |  |  |  |  |  |  |  |
| Poor | 0.000 | -0.500 | 0.000 | -0.001 | -0.03*** | -0.500 | 0.015 | -0.036 | -0.03*** | -0.50 | 0.013 | -0.137 |
| Middle | -0.009 | 0.000 | 0.000 | 0.000 | -0.05*** | 0.000 | 0.000 | 0.000 | -0.03*** | 0.00 | 0.000 | 0.000 |
| Rich | -0.024** | 0.500 | -0.012 | 0.129 | -0.07*** | 0.501 | -0.035 | 0.084 | -0.04*** | 0.50 | -0.021 | 0.220 |
| Richest | -0.05*** | 1.000 | -0.051 | 0.550 | -0.10*** | 1.000 | -0.096 | 0.228 | -0.08*** | 1.00 | -0.080 | 0.858 |
| Household Size (ref: < 6 members) | | | | |  |  |  |  |  |  |  |  |
| Member (6 to 11) | -0.013* | -0.153 | 0.002 | -0.021 | -0.012*** | -0.118 | 0.001 | -0.003 | -0.04*** | -0.198 | 0.008 | -0.088 |
| Member (>11) | -0.023** | -0.329 | 0.008 | -0.081 | -0.012 | -0.134 | 0.002 | -0.004 | -0.08*** | -0.353 | 0.027 | -0.291 |
| Household Composition |  |  |  |  |  |  |  |  |  |  |  |  |
| At least one child (<6 yrs) | -0.007 | -0.341 | 0.002 | -0.024 | -0.009* | -0.188 | 0.002 | -0.004 | 0.005 | -0.343 | -0.002 | 0.017 |
| At least one ember (>=65 yrs) | 0.009 | -0.061 | -0.001 | 0.006 | 0.009** | -0.015 | 0.000 | 0.000 | 0.006 | -0.037 | 0.000 | 0.002 |
| At least one employed member | -0.017 | -0.312 | 0.005 | -0.056 | -0.007 | -0.188 | 0.001 | -0.003 | 0.002 | -0.302 | -0.001 | 0.008 |
| Household Head Characteristics | | | | |  |  |  |  |  |  |  |  |
| Age categories (ref: age<=34) | | | | |  |  |  |  |  |  |  |  |
| 35-44 | 0.002 | -0.033 | 0.000 | 0.001 | -0.02*** | -0.031 | 0.000 | -0.001 | -0.004 | -0.061 | 0.000 | -0.003 |
| 45-54 | 0.011 | 0.021 | 0.000 | -0.002 | -0.015*** | 0.037 | -0.001 | 0.001 | -0.003 | 0.049 | 0.000 | 0.002 |
| >54 | 0.013 | 0.005 | 0.000 | -0.001 | -0.005 | 0.017 | 0.000 | 0.000 | 0.001 | 0.061 | 0.000 | -0.001 |
| Educational Categories (ref: illiterate) | | | | |  |  |  |  |  |  |  |  |
| Primary | -0.036 | -0.055 | 0.002 | -0.021 | 0.001 | -0.077 | 0.000 | 0.000 | -0.008 | -0.083 | 0.001 | -0.007 |
| Metric | -0.040 | 0.226 | -0.009 | 0.097 | 0.007 | 0.106 | 0.001 | -0.002 | -0.007 | 0.201 | -0.001 | 0.014 |
| Graduation | -0.035 | 0.485 | -0.017 | 0.183 | 0.017*** | 0.285 | 0.005 | -0.012 | -0.015 | 0.487 | -0.007 | 0.078 |
| Postgrad | -0.055 | -0.302 | 0.017 | -0.176 | 0.011 | 0.447 | 0.005 | -0.011 | -0.048** | 0.672 | -0.032 | 0.342 |
| Gender (ref : male) | | | | |  |  |  |  |  |  |  |  |
| Female | 0.009 | -0.178 | -0.002 | 0.017 | -0.004 | -0.135 | 0.001 | -0.001 | -0.03*** | 0.193 | -0.005 | 0.059 |
| Marital Status (ref : unmarried) | | | | |  |  |  |  |  |  |  |  |
| Married | 0.001 | -0.117 | 0.000 | 0.001 | 0.003 | -0.081 | 0.000 | 0.000 | -0.026 | -0.114 | 0.003 | -0.032 |
| Employment (ref: unemployed) | | | | |  |  |  |  |  |  |  |  |
| Employed (HH) | 0.001 | -0.068 | 0.000 | 0.001 | -0.011*** | -0.085 | 0.001 | -0.002 | -0.03*** | -0.114 | 0.003 | -0.032 |
| Region (ref: rural) | | | | |  |  |  |  |  |  |  |  |
| Urban | -0.027 | 0.258 | -0.007 | 0.075 | -0.005 | 0.230 | -0.001 | 0.002 | 0.013* | 0.389 | 0.005 | -0.054 |
| Province (ref: KP) | | | | |  |  |  |  |  |  |  |  |
| Punjab | -0.03*** | -0.054 | 0.002 | -0.018 | -0.011 | 0.059 | -0.001 | 0.002 | -0.03*** | 0.211 | -0.006 | 0.063 |
| Sindh | 0.05*** | -0.049 | -0.002 | 0.025 | -0.026*** | -0.052 | 0.001 | -0.003 | -0.10*** | -0.060 | 0.006 | -0.065 |
| Balochistan | -0.10** | -0.470 | 0.048 | -0.511 | -0.034* | -0.140 | 0.005 | -0.011 | -0.10*** | -0.376 | 0.037 | -0.395 |
| Residual | . | . | -0.077 | 0.827 | . | . | -0.326 | 0.777 | . | . | -0.038 | 0.406 |
| CI | . | . | -0.094 | 1.000 | . | . | -0.420 | 1.000 | . | . | -0.093 | 1.000 |
| Standard errors in parentheses; *** p<0.01, ** p<0.05, * p<0.1 represents significance at 1%, 5% and 10% level of significance. ME, CI, are Marginal Effects and Concentration Index of each factor. Absolute and relative are absolute and relative contribution of each factor to inequality. | | | | | | | | | | | | |

**Table A4: Decomposition of inequalities in catastrophic health expenditures (40% threshold, Wagstaff Method)**

|  | 2007-08 | | | | 2011-12 | | | | 2018-19 | | | |
| --- | --- | --- | --- | --- | --- | --- | --- | --- | --- | --- | --- | --- |
| (1) | (2) | (3) | (4) | (5) | (6) | (7) | (8) | (9) | (11) | (12) | (13) | (14) |
|  | ME | CI | absolute | relative | ME | CI | absolute | relative | ME | CI | absolute | relative |
| Socioeconomic Quintiles (ref: poorest) | | | | |  |  |  |  |  |  |  |  |
| Poor | -0.001 | -0.500 | 0.000 | -0.003 | -0.02*** | -0.500 | 0.008 | -0.015 | -0.009*** | -0.500 | 0.004 | -0.021 |
| Middle | -0.008 | 0.000 | 0.000 | 0.000 | -0.02*** | 0.000 | 0.000 | 0.000 | -0.013*** | 0.000 | 0.000 | 0.000 |
| Rich | -0.018*** | 0.500 | -0.009 | 0.057 | -0.04*** | 0.501 | -0.019 | 0.036 | -0.024*** | 0.500 | -0.012 | 0.057 |
| Richest | -0.039*** | 1.000 | -0.039 | 0.249 | -0.07*** | 1.000 | -0.069 | 0.130 | -0.040*** | 1.000 | -0.040 | 0.187 |
| Household Size (ref: < 6 members) | | | | |  |  |  |  |  |  |  |  |
| Member (6 to 11) | -0.008* | -0.008 | -0.153 | 0.001 | -0.003 | -0.118 | 0.000 | -0.001 | -0.015*** | -0.198 | 0.003 | -0.014 |
| Member (>11) | -0.019** | -0.019 | -0.329 | 0.006 | -0.003 | -0.134 | 0.000 | -0.001 | -0.037 | -0.353 | 0.013 | -0.062 |
| Household Composition |  |  |  |  |  |  |  |  |  |  |  |  |
| At least one child (<6 yrs) | 0.001 | 0.001 | -0.341 | 0.000 | -0.005 | -0.188 | 0.001 | -0.002 | 0.004*** | -0.343 | -0.001 | 0.006 |
| At least one member (>=65 yrs) | 0.008 | 0.008 | -0.061 | 0.000 | 0.002 | -0.015 | 0.000 | 0.000 | 0.001 | -0.037 | 0.000 | 0.000 |
| At least one employed member | -0.016 | -0.016 | -0.312 | 0.005 | -0.011 | -0.188 | 0.002 | -0.004 | -0.010* | -0.302 | 0.003 | -0.014 |
| Household Head Characteristics | | | | |  |  |  |  |  |  |  |  |
| Age categories (ref: age<=34) | | | | |  |  |  |  |  |  |  |  |
| 35-44 | -0.001 | -0.033 | 0.000 | 0.000 | -0.004 | -0.031 | 0.000 | 0.000 | -0.003 | -0.061 | 0.000 | -0.001 |
| 45-54 | 0.014** | 0.021 | 0.000 | -0.002 | -0.005 | 0.037 | 0.000 | 0.000 | -0.007* | 0.049 | 0.000 | 0.002 |
| >54 | 0.012* | 0.005 | 0.000 | 0.000 | 0.005 | 0.017 | 0.000 | 0.000 | -0.002 | 0.061 | 0.000 | 0.001 |
| Educational Categories (ref: illiterate) | | | | |  |  |  |  |  |  |  |  |
| Primary | -0.028 | -0.055 | 0.002 | -0.010 | 0.004 | -0.077 | 0.000 | 0.001 | -0.003 | -0.083 | 0.000 | -0.001 |
| Metric | -0.027 | 0.226 | -0.006 | 0.039 | 0.006* | 0.106 | 0.001 | -0.001 | -0.002 | 0.201 | 0.000 | 0.002 |
| Graduation | -0.027 | 0.485 | -0.013 | 0.083 | 0.007 | 0.285 | 0.002 | -0.004 | -0.004 | 0.487 | -0.002 | 0.010 |
| Postgrad | -0.031 | -0.302 | 0.009 | -0.059 | -0.001 | 0.447 | -0.001 | 0.001 | -0.004 | 0.672 | -0.002 | 0.011 |
| Gender (ref : male) | | | | |  |  |  |  |  |  |  |  |
| Female | -0.005 | -0.178 | 0.001 | -0.006 | -0.005 | -0.135 | 0.001 | -0.001 | -0.008 | 0.193 | -0.001 | 0.007 |
| Marital Status (ref : unmarried) | | | | |  |  |  |  |  |  |  |  |
| Married | 0.001 | -0.117 | 0.000 | 0.001 | 0.010** | -0.081 | -0.001 | 0.001 | 0.010** | -0.135 | -0.001 | 0.006 |
| Employment (ref: unemployed) | | | | |  |  |  |  |  |  |  |  |
| Employed (HH) | 0.005 | -0.068 | 0.000 | 0.002 | 0.002 | -0.085 | 0.000 | 0.000 | -0.006 | -0.114 | 0.001 | -0.003 |
| Region (ref: rural) | | | | |  |  |  |  |  |  |  |  |
| Urban | -0.020*** | 0.258 | -0.005 | 0.033 | -0.009** | 0.230 | -0.002 | 0.004 | -0.005 | 0.389 | -0.002 | 0.009 |
| Province (ref: KP) | | | | |  |  |  |  |  |  |  |  |
| Punjab | -0.019** | -0.054 | 0.001 | -0.006 | -0.004 | 0.059 | 0.000 | 0.000 | -0.008*** | 0.211 | -0.002 | 0.008 |
| Sindh | 0.021*** | -0.049 | -0.001 | 0.006 | -0.02*** | -0.052 | 0.001 | -0.002 | -0.021*** | -0.060 | 0.001 | -0.006 |
| Balochistan | -0.053*** | -0.470 | 0.025 | -0.158 | -0.010 | -0.140 | 0.001 | -0.003 | -0.028*** | -0.376 | 0.010 | -0.049 |
| Residual | . | . | -0.134 | 0.849 | . | . | -0.455 | 0.858 | . | . | -0.185 | 0.866 |
| CI | . | . | -0.158 | 1.000 | . | . | -0.530 | 1.000 | . | . | -0.213 | 1.000 |
| Standard errors in parentheses; *** p<0.01, ** p<0.05, * p<0.1 represents significance at 1%, 5% and 10% level of significance. ME, CI, are Marginal Effects and Concentration Index of each factor. Absolute and relative are absolute and relative contribution of each factor to inequality. | | | | | | | | | | | | |
